# Author Correction: Chirality flips of skyrmion bubbles

**DOI:** 10.1038/s41467-022-35221-5

**Published:** 2022-12-01

**Authors:** Yuan Yao, Bei Ding, Jinjing Liang, Hang Li, Xi Shen, Richeng Yu, Wenhong Wang

**Affiliations:** 1 Beijing National Laboratory for Condensed Matter Physics, Institute of Physics, Chinese Academy of Sciences, 100190 Beijing, China; 2 University of Chinese Academy of Sciences, 100049 Beijing, China; 3 Songshan Lake Materials Laboratory, 523808 Dongguan, Guangdong China

**Keywords:** Magnetic properties and materials, Magnetic properties and materials, Spintronics

Correction to: *Nature Communications* 10.1038/s41467-022-33700-3, published online 11 October 2022

The original version of this article contained an error in Table 1 of the manuscript. The original version of this table was


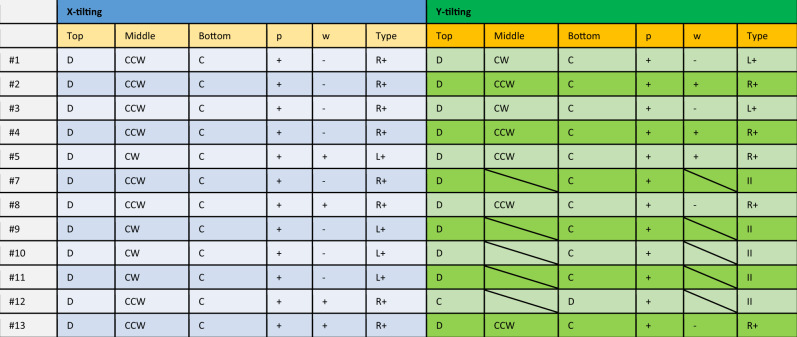


Which contained errors in the following cells, highlighted below:


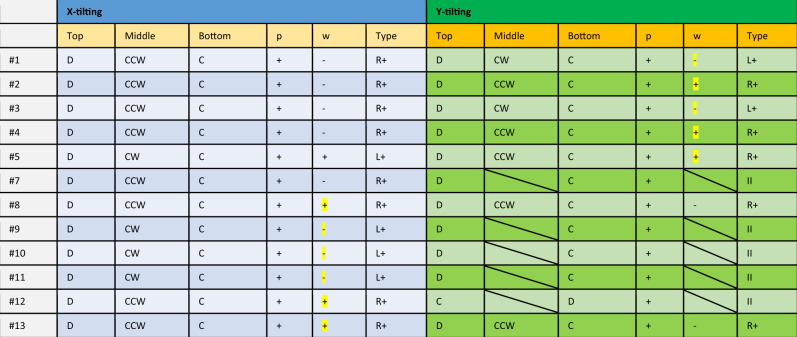


The sign of “w” should be “+” if the rotation in middle is Clockwise, “CW”, and be “−” if the rotation is Counter Clockwise, “CCW”. The corrected form of Table 1 is:


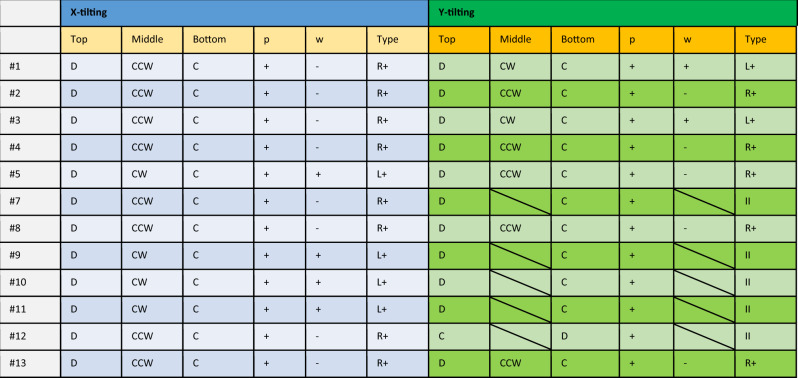


Table 1 in the article has been updated with the corrected version.

