# Supplementary material for: Chirality flips of skyrmion bubbles
Source: Nat Commun. 2022 Oct 11;13:5991. doi: 10.1038/s41467-022-33700-3 (PMC9553972; doi:10.1038/s41467-022-33700-3)
Supplement: Supplementary file 1 — Supplementary Information [file 41467_2022_33700_MOESM1_ESM.pdf]

Supplementary Information

Chirality flips of skyrmion bubble

Yuan Yao[1,4*], Bei Ding[1,4], Jinjing Liang[1,3], Hang Li[1], Xi Shen[1], Richeng Yu[1], Wenhong Wang[1,2,3*]

[1]Beijing National Laboratory for Condensed Matter Physics, Institute of Physics, Chinese Academy of Sciences, Beijing 100190, China

[2] Songshan Lake Materials Laboratory, Dongguan, Guangdong 523808, China

[3]University of Chinese Academy of Sciences, Beijing 100049, China

[4]These authors contributed equally: Yuan Yao and Bei Ding

Corresponding Author

*E-mail: yaoyuan@iphy.ac.cn
wenhong.wang@iphy.ac.cn

1. Transport of intensity equation (TIE): principle and regularization parameter[1]

The TIE was established by Teague[2] in 1982 to relate the intensity variation and phase of a wave or to solve the inverse problem. The general expression of the image intensity and the wave phase at position (x,y,z) is

$$-k\frac{\partial I(x,y,z)}{\partial z} = \nabla_{xy}\cdot[I(x,y,z)\nabla_{xy}\varphi(x,y,z)] \quad (1)$$

where the wave function is $U(x,y,z) = \sqrt{I(x,y,z)}\exp[i\varphi(x,y,z)]$, propagating along the z direction; k represents its wave number, and ∇_{xy} is the gradient operator in the x-y plane, which is perpendicular to the propagation direction. This equation can be derived from several frameworks, such as the paraxial wave model or Fresnel diffraction model. supplementary Equation 1 reveals an approach to retrieve the phase of a wave from its image contrast at different positions. Obviously, obtaining the solution of supplementary Equation 1 is not easy because it is a second-order elliptic partial differential equation. With the auxiliary function[3]

$$I(x,y,z)\nabla_{xy}\varphi(x,y,z) = \nabla_{xy}\phi(x,y) \quad (2)$$

supplementary Equation 1 can be transformed into

$$\varphi(x,y,z_0) = -k\nabla_{x,y}^{-2}\left\{\nabla_{x,y}\left[\frac{\nabla_{x,y}\nabla_{x,y}^{-2}\left(\frac{\partial I(x,y,z)}{\partial z}\right)}{I(x,y,z_0)}\right]\right\} \quad (3)$$

where $\nabla_{x,y}^{-2}$ is the inverse Laplacian operator. Note that the validity of the auxiliary function requires current conservation of the wave phase, which is satisfied in Lorentz transmission electron microscopy (LTEM) experiments since the scattering angle is very small and the electron dose is almost constant in the images. No other assumptions are required. However, the differential and normalization in supplementary Equation 3 remove the influence of the amplitude of the wave. For our work, there is no singularity in the wave phase. The simulated phase image of a skyrmion bubble also confirms this point (supplementary Figure 1). Thus, supplementary Equation 2 is satisfied here. The situation for an electron vortex beam containing a phase singularity has been addressed by Lubk et al[4]. A rapid method to solve supplementary Equation 3 is to transform it to reciprocal space, and $\nabla_{x,y}^{-2}[f(x,y)]$ can be substituted by $\mathcal{F}^{-1}\left\{\frac{-\mathcal{F}[\cdot]}{|\mathbf{q}|^2}\right\}$ ($\mathcal{F}$ and $\mathcal{F}^{-1}$ are the Fourier transform and inverse transform, respectively, and $\mathbf{q}$ is the frequency in reciprocal space). To avoid divergence, a regularization parameter q_0 should be appended: $\frac{-|\mathbf{q}|^2}{(|\mathbf{q}|^2+{q_0}^2)^2}$. This regularization is an effective Tikhonov-type filter that can suppress the low frequency noise but may exaggerate the contribution from high spatial frequencies (supplementary Figure 2 and Figure 8).[5, 6] This solution is also sensitive to the boundary condition, which is difficult to measure. We select the "Padding" approach proposed by Volkov et al[7] in the implemented TIE calculation.

a) b)

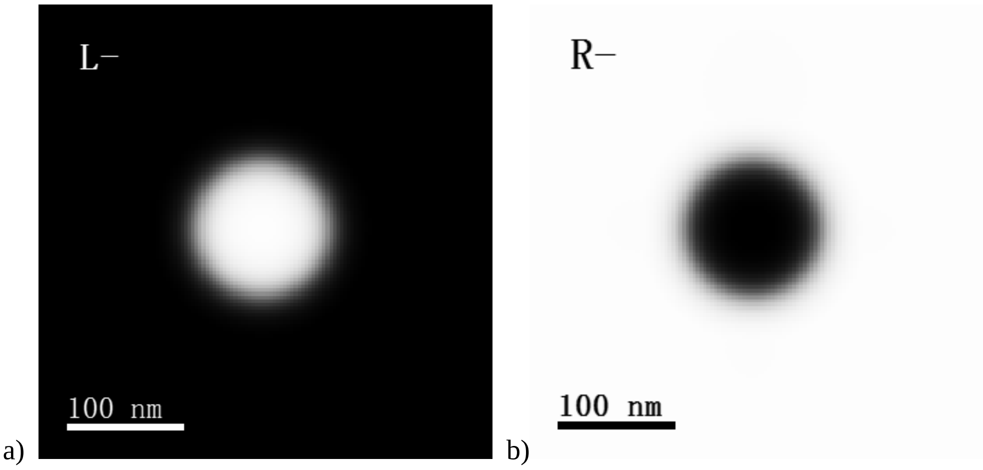

Supplementary Figure 1 Phases of waves exiting from the a) L- and b) R- skyrmion models.

a) 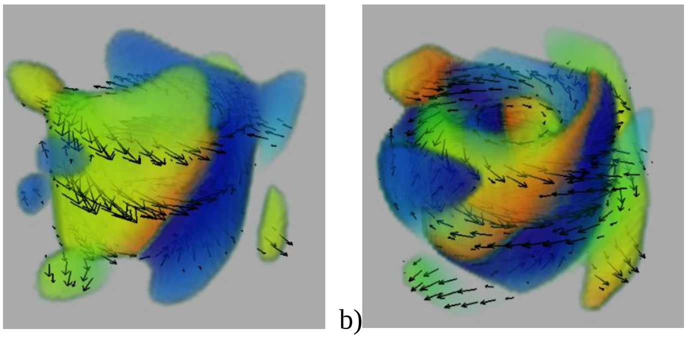 b)

Supplementary Figure 2 Reconstructed 3D B_x-B_y features for R+ skyrmions with different regularizations q_0. a) $q_0 = 1 \times 10^{-3}$ nm^{-1}, b) $q_0 = 5 \times 10^{-3}$ nm^{-1}. A larger q_0 can obviously enhance the magnetic structure feature but introduces some artifacts.

2. Magnetic structure simulation: magnetization, induction and LTEM images

Micromagnetic simulations were carried out by using the 3D object oriented micromagnetic framework (OOMMF) with the parameters used in a previous report.[8] supplementary Figure 3 demonstrates the magnetization configurations of four Type-I skyrmions with different chiralities, while supplementary Figure 4 shows the configuration for a Type-II bubble.

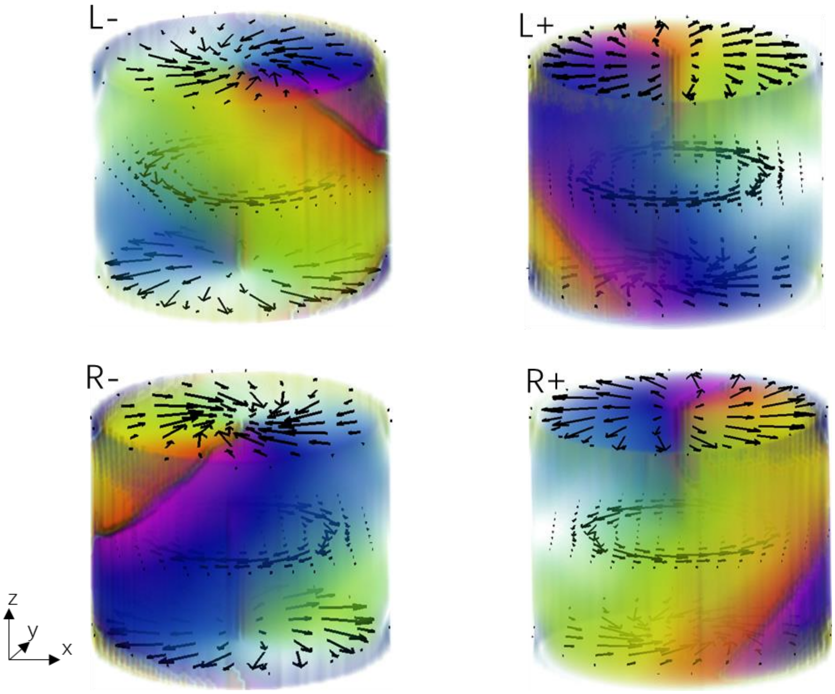

Supplementary Figure 3 Rendered 3D structure of the in-plane components of various Type-I bubbles.

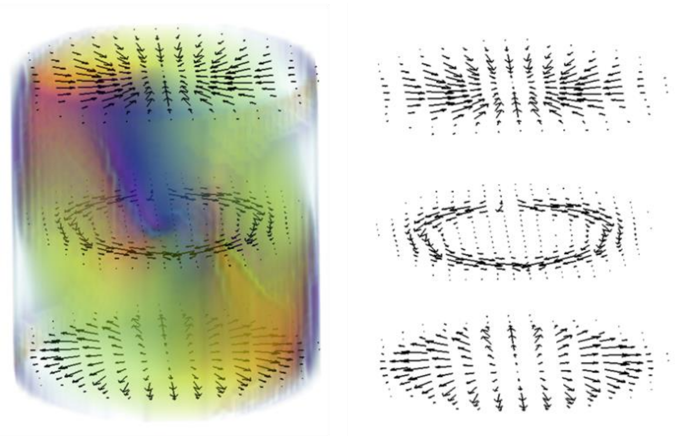

Supplementary Figure 4 Rendered 3D structure of the in-plane components of Type-II bubbles.

The OOMMF can offer the magnetization distribution of skyrmion bubbles, but the LTEM image contrast is determined by the projection of the magnetic vector potential $\mathbf{A(r)}$ or the integral of $A_z(\mathbf{r})$ along the electron propagation direction (if only considering the contribution from the magnetic field).

$$\varphi(\mathbf{r}) = -\frac{2\pi e}{h}\oint \mathbf{A(r)}\cdot d\mathbf{z} = -\frac{2\pi e}{h}\int A_z(\mathbf{r})dz \tag{4}$$

The vector potential $\mathbf{A(r)}$ should be calculated from magnetization matrix $\mathbf{M(r)}$:[9]

$$\mathbf{A(r)} = \frac{\mu_0}{4\pi}\int \mathbf{M(r')}\times\frac{\mathbf{r}-\mathbf{r'}}{|\mathbf{r}-\mathbf{r'}|}d^3\mathbf{r'} \tag{5}$$

We adapt the calculation in the frequency domain:

$$\mathbf{A(q)} = -\frac{i\mu_0}{\mathbf{q}^2}\mathbf{M(q)}\times\mathbf{q} \tag{6}$$

Then, $\mathbf{A(r)}$ is obtained by the inverse Fourier transform. The curl of $\mathbf{A(r)}$ results in the magnetic induction $\mathbf{B(r)}$:

$$\mathbf{B(r)} = \nabla\times\mathbf{A(r)} \tag{7}$$

The advantage of this method is that the demagnetization and stray field are auto-consistent in the calculation, and the computing efficiency can be accelerated by a fast Fourier transform (FFT) algorithm. The magnetization was assumed to be a pure phase object, and only the phase of the exit wave contributed to the image contrast. The obtained vector potential matrix was tilted around the given axis to produce the phase shift of the exit wave based on supplementary Equation 4. LTEM images were generated by convoluting the exit wave with the contrast transfer function, which involved the electron energy (200 keV), Cs (5 m), defocus (0 and ±300 μm) and dispersion envelope

(100 Å). supplementary Figure 5 shows some simulated LTEM images.

a) 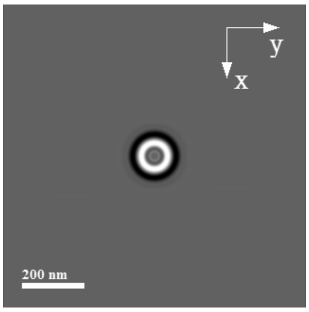

b) 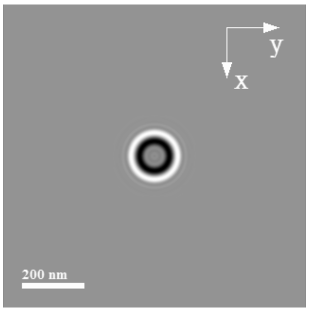

c) 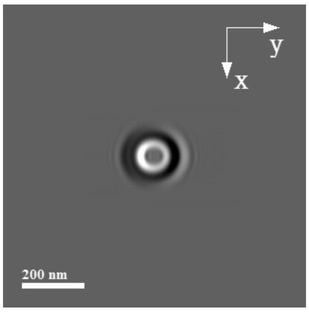

d) 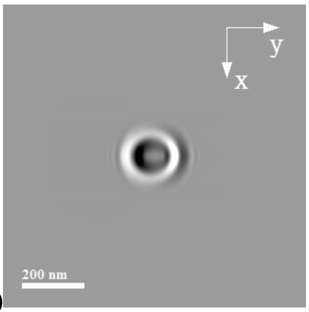

Supplementary Figure 5 Simulated LTEM images for an R+ skyrmion: a) and b) underfocus and overfocus without tilting; c) and d) underfocus and overfocus with tilting -30° around the x-axis.

3. Raw experimental images of tilt series

Raw images of x-tilt and y-tilt series were acquired with a JEOL 2100F at a defocus of 300 μm for over- and under-focused images. The exposure time was fixed to 1 s for all images to ensure a uniform total dose.

a) 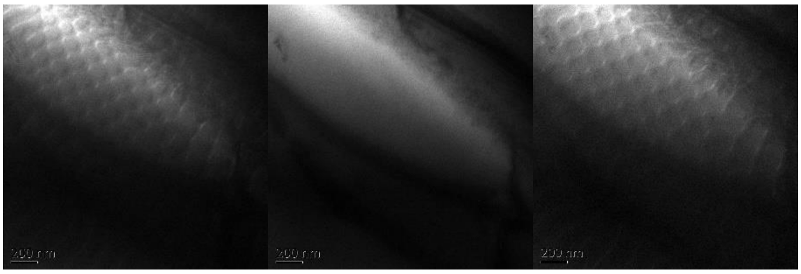

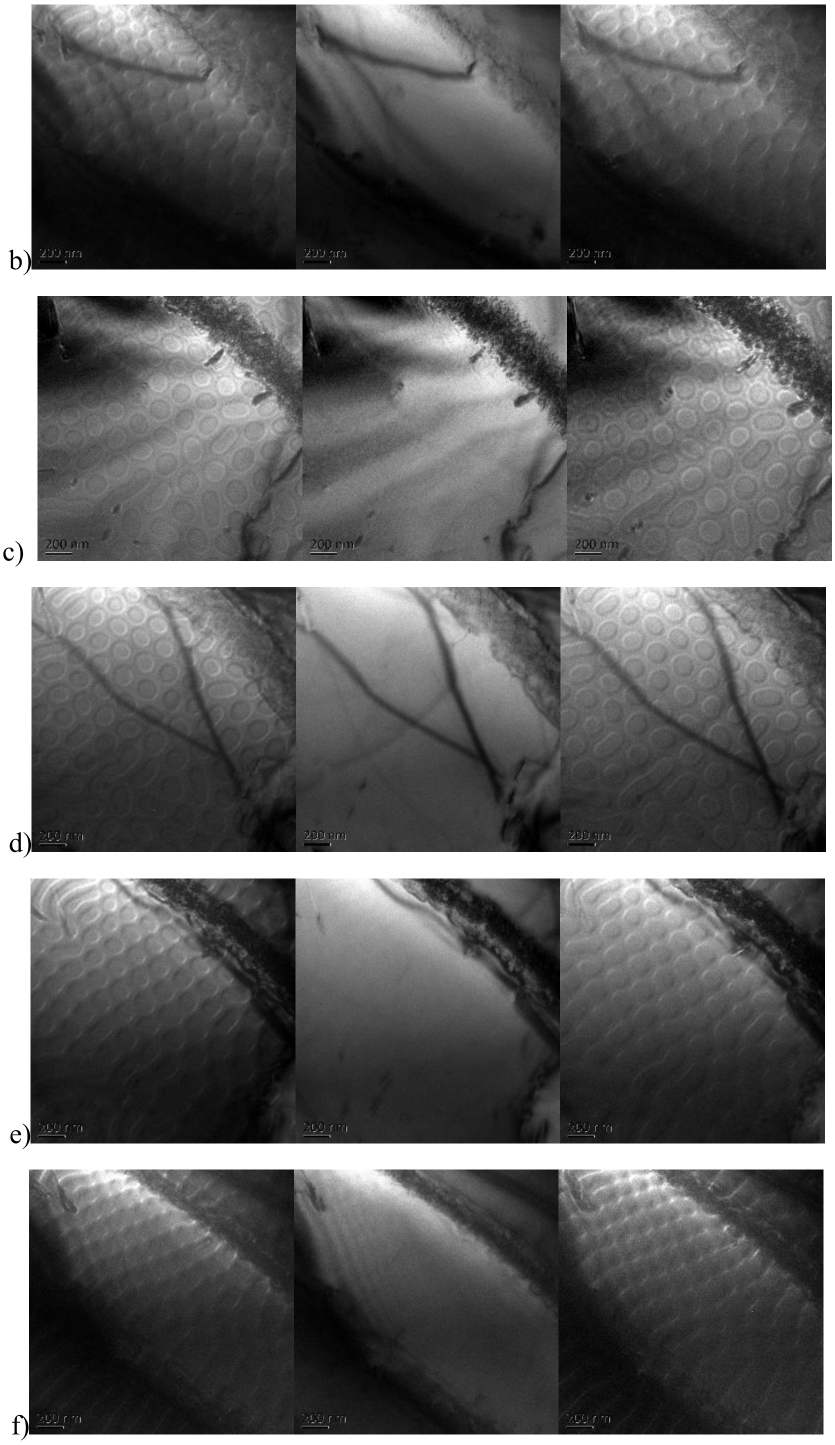

Supplementary Figure 6 Under focused, in focused and over focused images for x-tilt: a) -54.8°, b) -43.3°, c) 0.4°, d) 10.9°, e) 30.4°, and f) 52.1°.

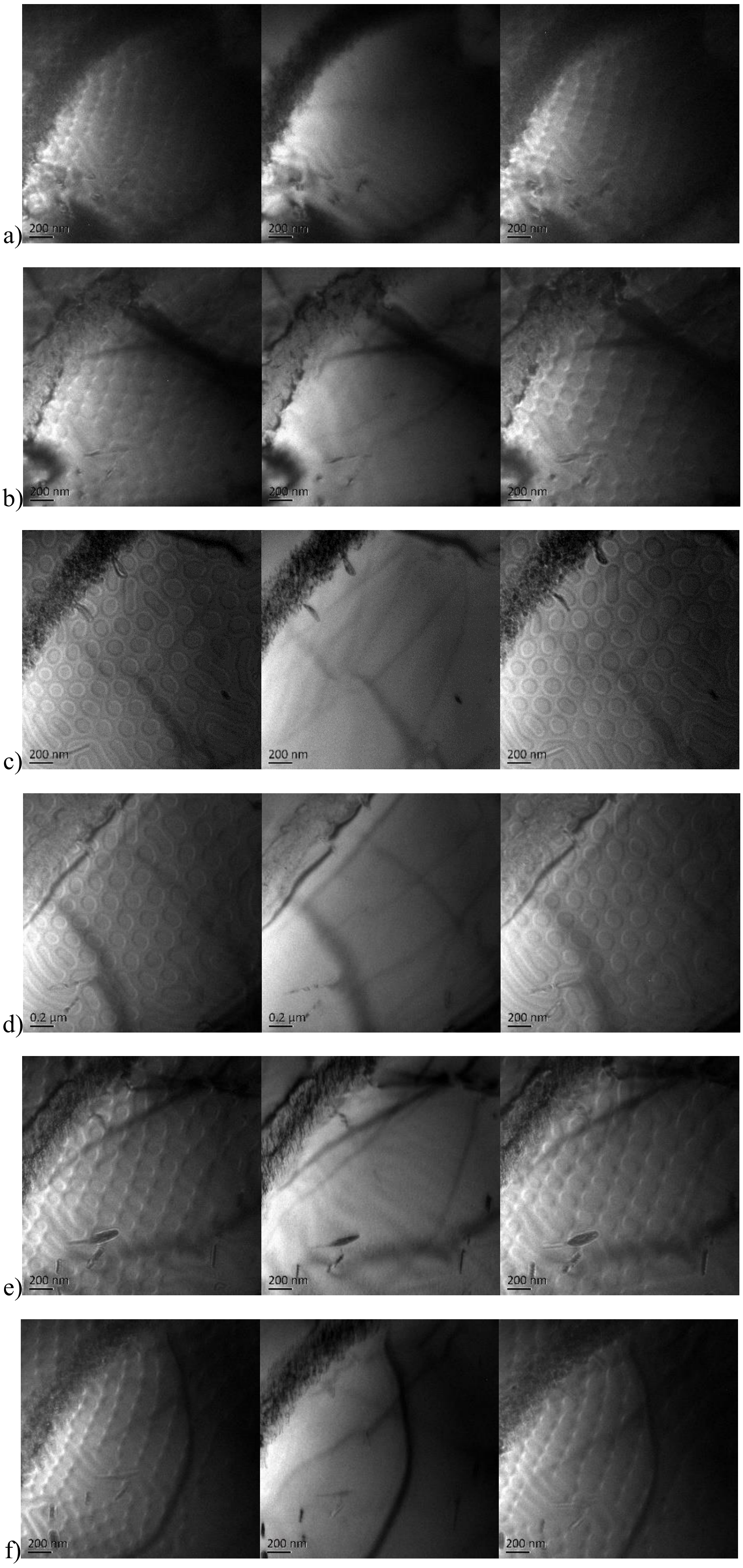

Supplementary Figure 7 Under focused, in focused and over focused images for y-tilt: a) -56.6°,

b) -42.7°, c) 1.2°, d) 16.3°, e) 33.9°, and f) 47.6°.

4. Reconstruction of 3D induction: simulated images and experimental images

The simulated and experimental images were input into the homemade TIE program to retrieve the phase image of the exit wave at different tilt angles. As mentioned above, the regularization parameter q_0 influences the retrieved phase, as demonstrated in supplementary Figure 8. $q_0=5\times10^{-3}$ nm^{-1} was chosen to recover the all-phase images to eliminate the possible artifacts (supplementary Figure 9 and Figure 10).

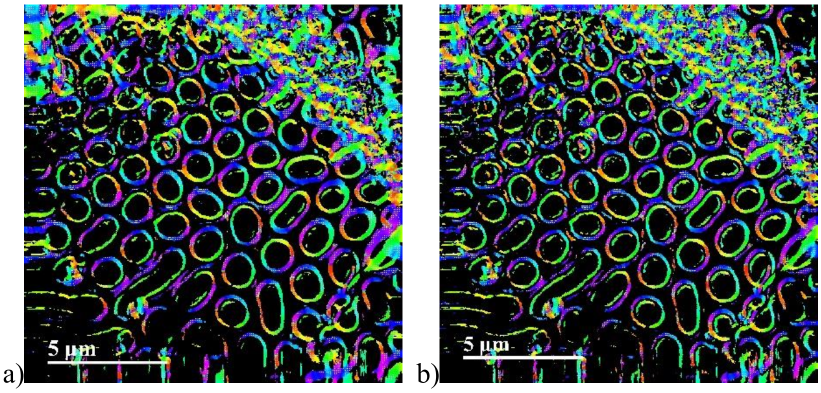

Supplementary Figure 8 In-plane components of induction retrieved with a) $q_0=5\times10^{-3}$ nm^{-1} and b) $q_0=7.5\times10^{-3}$ nm^{-1}.

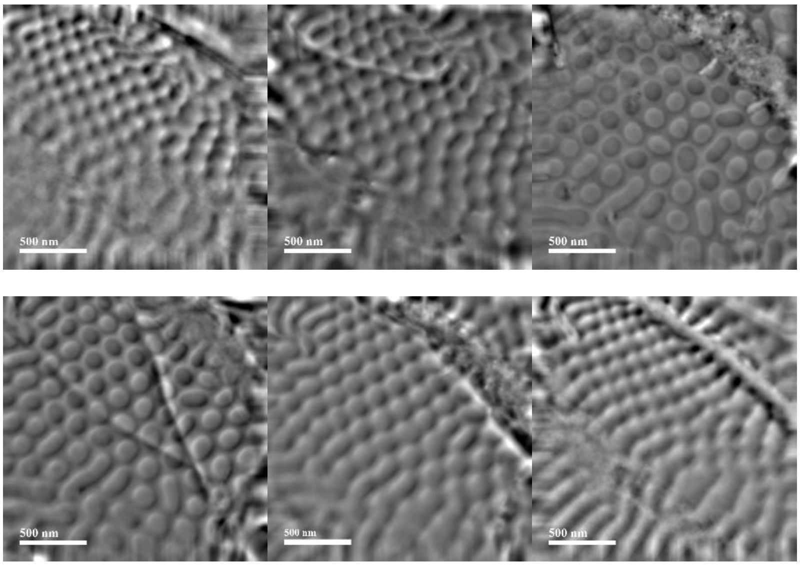

Supplementary Figure 9 Retrieved phase images for different x-tilt angles ($q_0=5\times10^{-3}$ nm^{-1}): a) -

54.8°, b) -43.3°, c) 0.4°, d) 10.9°, e) 30.4°, and f) 52.1°.

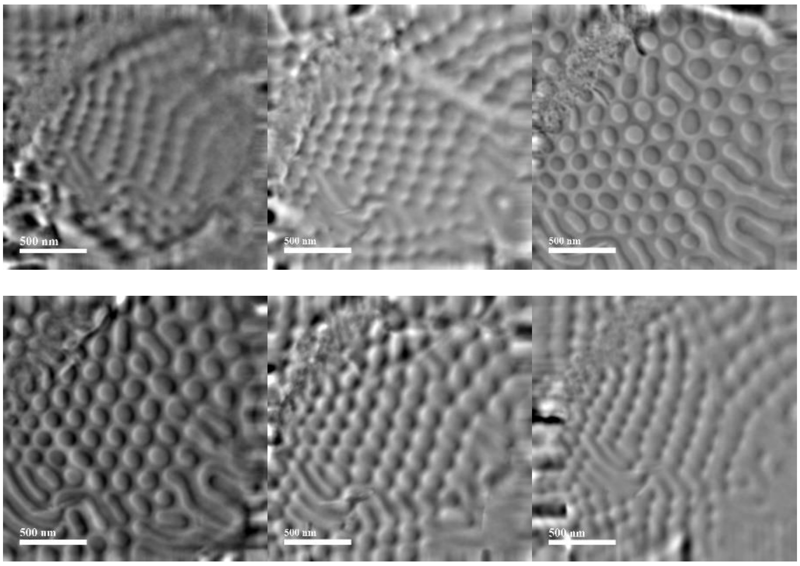

Supplementary Figure 10 Retrieved phase images for different y-tilt angles (q_0=5×10^{-3} nm^{-1}): a) -56.6°, b) -42.7°, c) 1.2°, d) 16.3°, e) 33.9°, and f) 47.6°.

The phase images, such as for x-tilting, were formed into one stack file and aligned with the "Image Alignment" function in Gatan Microscopy Suite (GMS). A "bandpass filter" with the default mode was employed to "automatically" register the images, followed by forward and backward corrections. After alignment, all phase images were merged into one image, and that image was transformed to Fourier space. The prolonged diffuse background of the autocorrelation region (center part) in the diffractogram indicated the tilt axis orientation[10], as shown in supplementary Figure 11. After the alignment, all phase images were rotated to make the tilt axis vertical or horizontal to the image boundary, facilitating the subsequent calculation of B_x or B_y components.

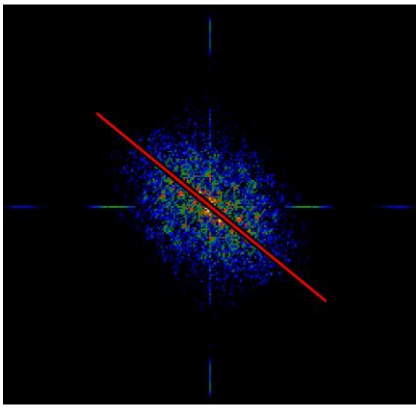

Supplementary Figure 11 Diffractogram of the merged phase image, where the dash denotes the orientation of the tilt axis.

B_x and B_y component mappings were calculated by differentiating the aligned phase images. The plug-in from Wolf et al[11] was used to complete the 3D reconstruction of Bx and By. The parameters for the reconstruction are shown in supplementary Figure 12. The simulation and experimental data share the same parameters to double check the fidelity of the results. supplementary Figure 13 shows the reconstructed in-plane induction of bubble #2.

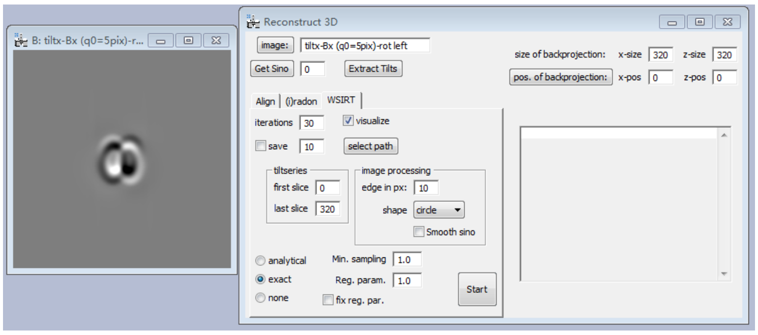

Supplementary Figure 12 GUI interface with the parameters for reconstruction processing.

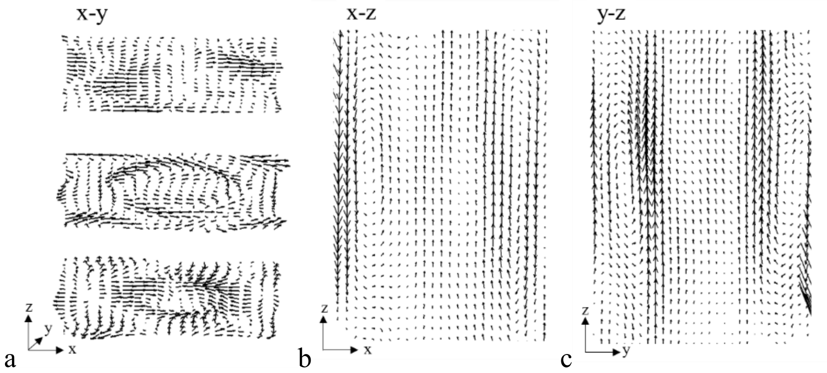

Supplementary Figure 13 Induction components within experiment bubble #2. a) Top, middle and bottom x-y sections; b) and c) moment components lying on the middle x-z section and y-z section.

5. 3D features of the reconstructed inductions for all bubbles

As described in the main text, the type of bubbles can be recognized from their 3D B_x or B_y component configurations. The bubbles marked in Figure 2 are shown in this section.

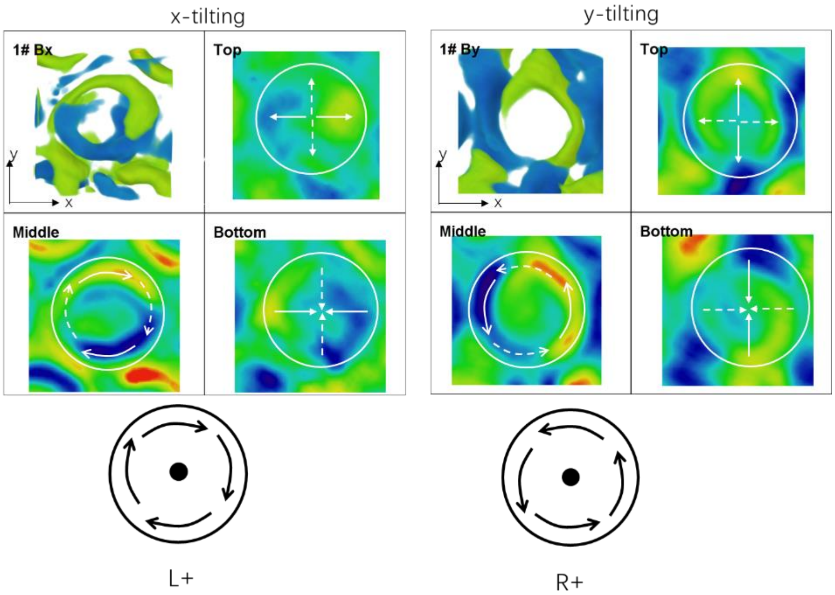
x-tilting
y-tilting
1# Bx
Top
Middle
Bottom
1# By
Top
Middle
Bottom
L+
R+

a

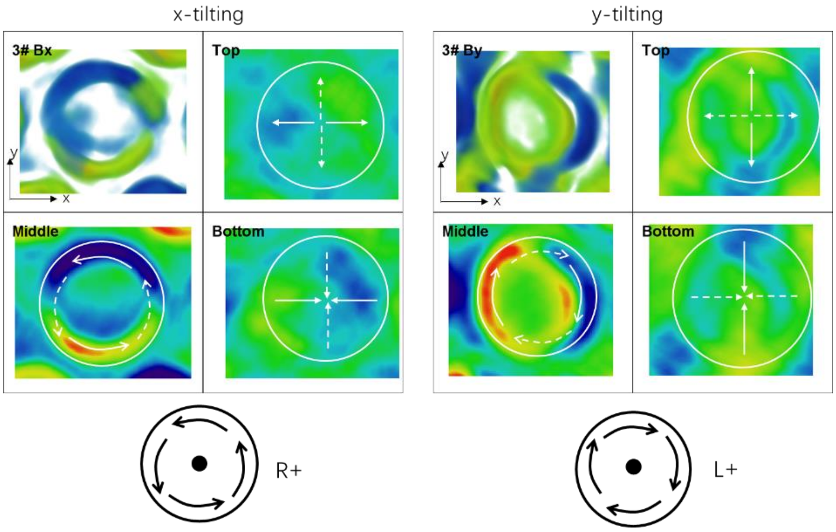
x-tilting
y-tilting
3# Bx
Top
Middle
Bottom
3# By
Top
Middle
Bottom
R+
L+

b

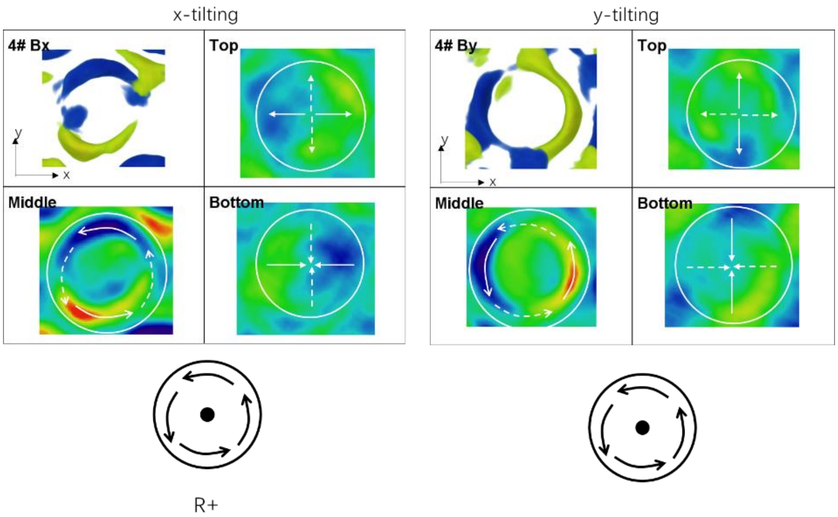
x-tilting
y-tilting
4# Bx
Top
Middle
Bottom
4# By
Top
Middle
Bottom
R+

c

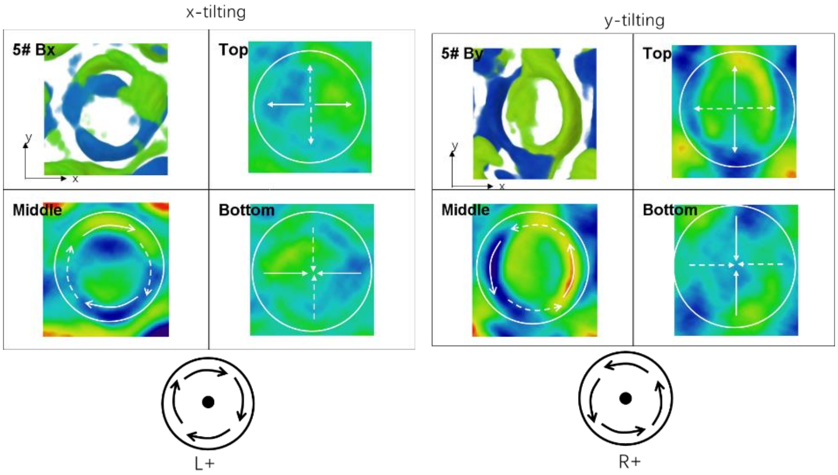
x-tilting
y-tilting
5# Bx
Top
Middle
Bottom
5# By
Top
Middle
Bottom
L+
R+

d

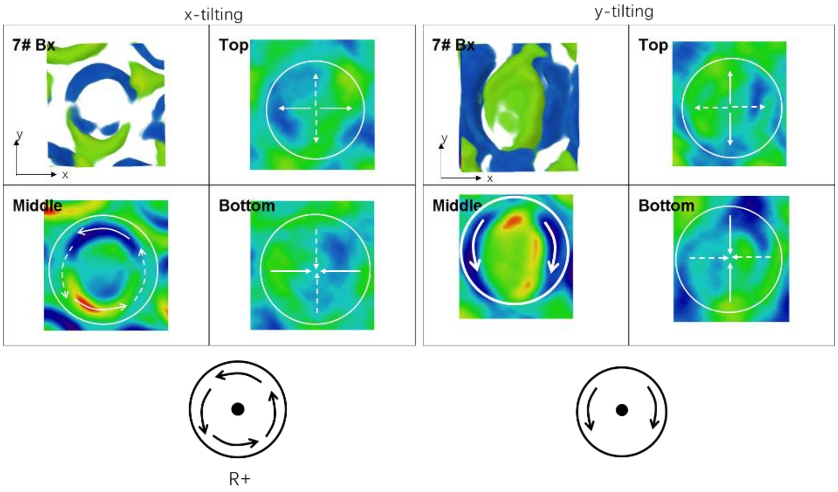
x-tilting
y-tilting
7# Bx
Top
Middle
Bottom
7# Bx
Top
Middle
Bottom
R+

e

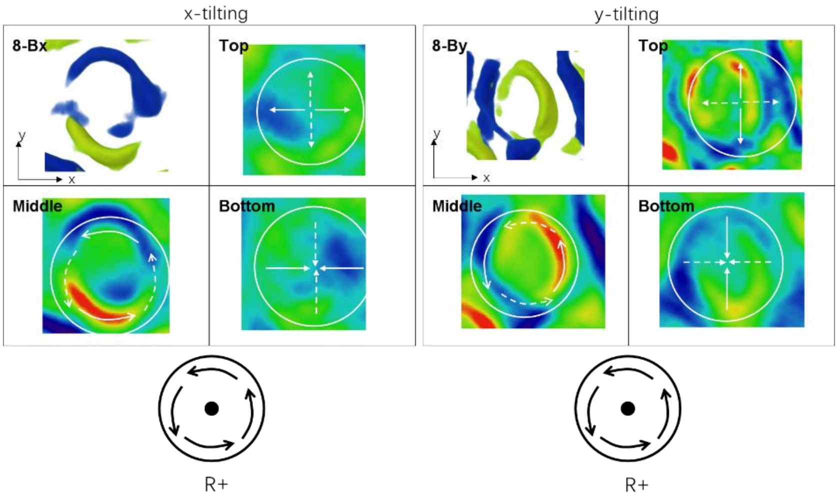
x-tilting
y-tilting
8-Bx
Top
Middle
Bottom
8-By
Top
Middle
Bottom
R+
R+

f

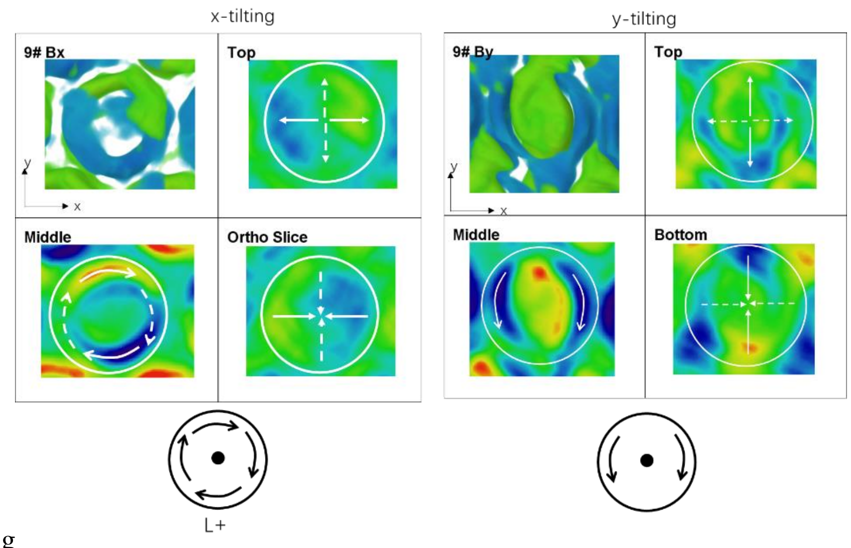

x-tilting
y-tilting
9# Bx
Top
Middle
Ortho Slice
9# By
Top
Middle
Bottom
L+

g

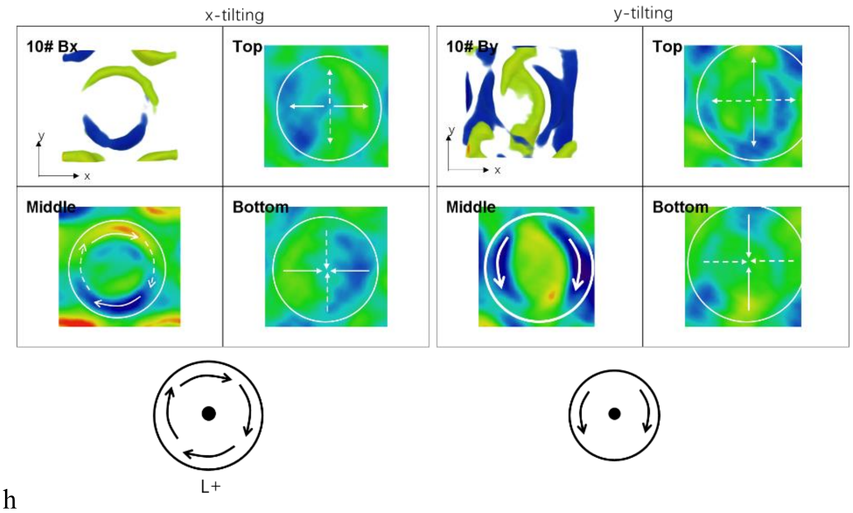

x-tilting
y-tilting
10# Bx
Top
Middle
Bottom
10# By
Top
Middle
Bottom
L+

h

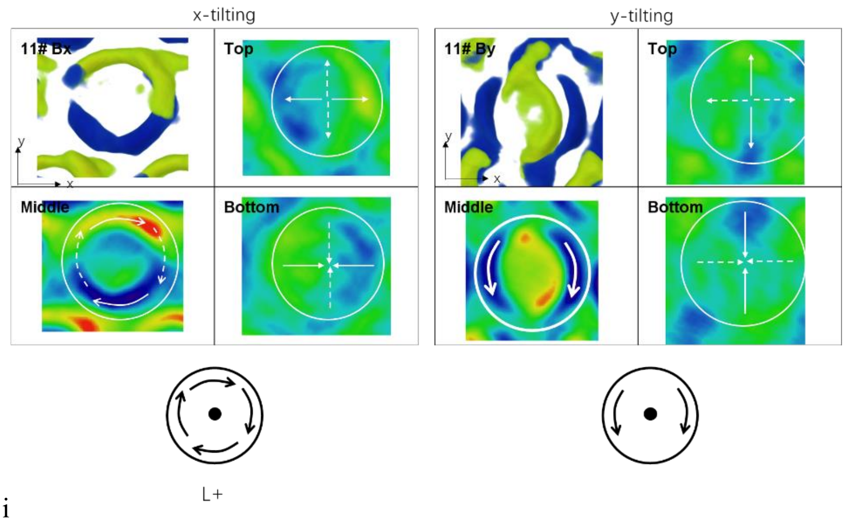

x-tilting
y-tilting
11# Bx
Top
Middle
Bottom
11# By
Top
Middle
Bottom
L+

i

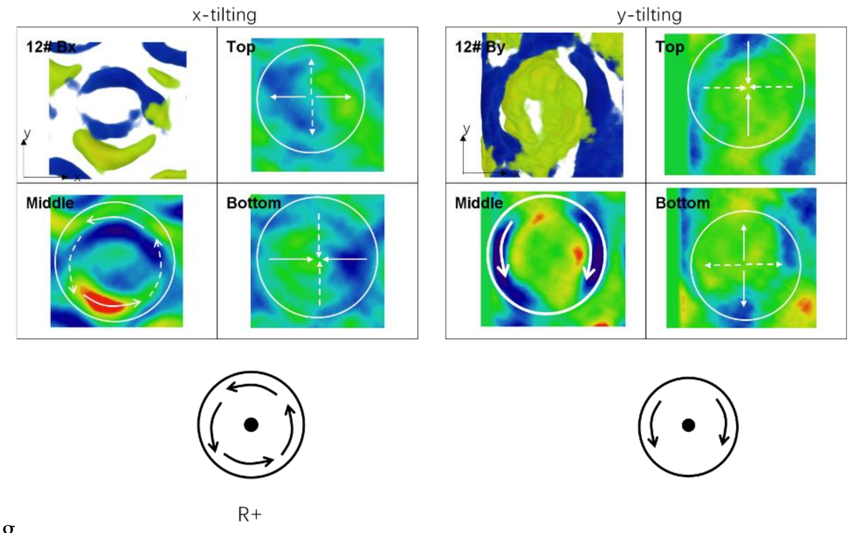

g

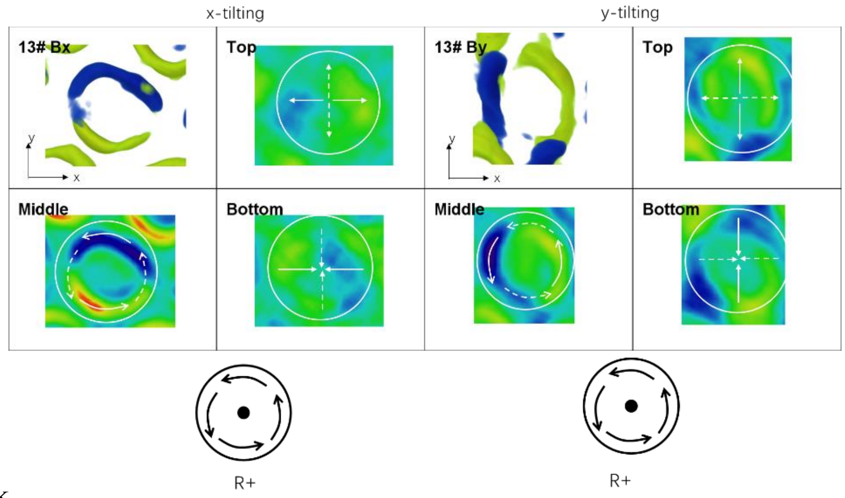

k

Supplementary Figure 14 Types of bubbles recognized from the 3D B_x or B_y component.

6. Influence of electron beam irradiation

The electron beam in LTEM can inject energy into the investigated region in the specimen. We assessed the stability of the bubble under a focused beam, which is stronger than normal illumination. After a curing time (twenty seconds), the bubble is stabilized in a new state until it is destroyed. In 3D data acquisition, the dose of the beam was weaker, but it also induced changes in some bubbles during the initial observation period.

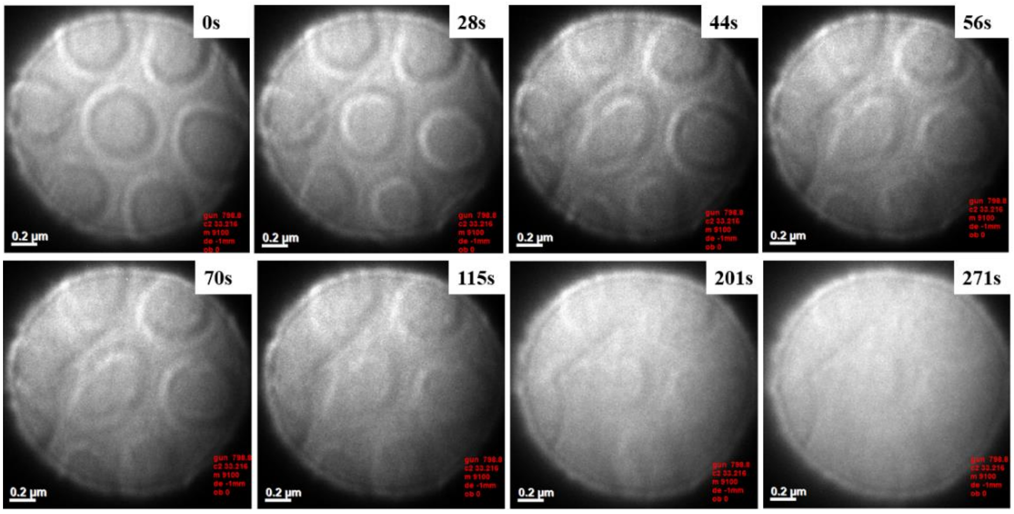

Supplementary Figure 15 Beam-induced changes in the bubbles.

7. Influence of the magnetic field in LTEM

The features of the bubbles did not change during the x-tilt or y-tilt data acquisition, which means that the chirality variation did not occur in the sample tilting but when the sample was operated outside of the LTEM system.

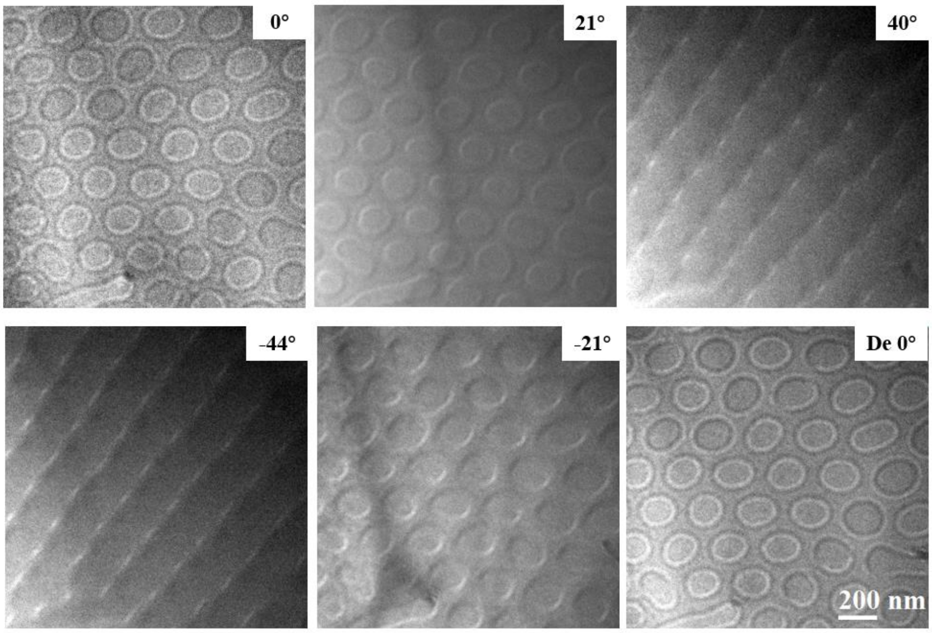

Supplementary Figure 16 The bubble features did not change during the x-tilt operation.

Supplementary Movie 1 B_x of the bubbles

Supplementary Movie 2 B_y of the bubbles

Supplementary Movie 3 B_x and B_y of bubble #2 and the R+ model

Supplementary Movie 4 Orientation mapping of the in-plane component of bubble #2 and the R+ model

Supplementary Movie 5 B_x and B_y of bubble #4

Supplementary Movie 6 x-tilt phase stack

Supplementary Movie 7 y-tilt phase stack

References

1. Zuo, C., Transport of intensity equation: a tutorial, Opt. Laser Eng., **135**, 106187 (2020)
2. Teague, M., Irradiance moments: their propagation and use for unique retrieval of phase, Journal of the Optical Society of America, **72**, 1199 (1982)
3. Paganin, D. and Nugent, K. A., Noninterferometric Phase Imaging with Partially Coherent Light, Phys. Rev. Lett., **80**, 2586 (1998)
4. Lubk. A. et al. Transport of Intensity Phase Retrieval of Arbitrary Wave Fields Including Vortices, Phys. Rev. Lett. **111**, 173902 (2013)
5. Yao, Y *et al.* Magnetic hard nanobubble: A possible magnetization structure behind the bi-skyrmion. *Appl. Phys. Lett.* **114**, 102404 (2019).
6. Cui, J. *et al.* Artifacts in magnetic spirals retrieved by transport of intensity equation (TIE). *J. Magn. Magn. Mater.* **454**, 304-313 (2018).
7. Volkov, V. V. et al. A new symmetrized solution for phase retrieval using the transport of intensity equation, Micron, **33**, 411-416 (2002)
8. Ding, B. *et al.* Manipulating Spin Chirality of Magnetic Skyrmion Bubbles by In-Plane Reversed Magnetic Fields in $(Mn_{1-x}Ni_x)_{65}Ga_{35}$(x = 0.45) Magnet. *Phys. Rev. Appl.* **12**, 054060 (2019)
9. Beleggia, M. and Zhu, Y., Electron-optical phase shift of magnetic nanoparticles I. Basic concepts, Philos. Mag., **83**, 1045- 1057 (2003)
10. Renken, C. et al. Markerless Alignment: Bridging the Gap Between Theory and Practice. Microsc. Microanal. **9**, 1170-1171, (2003).
11. https://wwwpub.zih.tu-dresden.de/~dwolf/
