# Supplementary material for: Chirality flips of skyrmion bubbles
Source: Nat Commun. 2022 Oct 11;13:5991. doi: 10.1038/s41467-022-33700-3 (PMC9553972; doi:10.1038/s41467-022-33700-3)
Supplement: Supplementary file 2 — Description of Additional Supplementary Files [file 41467_2022_33700_MOESM2_ESM.pdf]

Title: Supplementary Movie 1
Description: Bx of the bubbles

Title: Supplementary Movie 2
Description: By of the bubbles

Title: Supplementary Movie 3
Description: Bx and By of bubble #2 and the R+ model

Title: Supplementary Movie 4
Description: Orientation mapping of the in-plane component of bubble #2 and the R+ model

Title: Supplementary Movie 5
Description: Bx and By of bubble #4

Title: Supplementary Movie 6
Description: x-tilt phase stack

Title: Supplementary Movie 7
Description: y-tilt phase stack
